# Perceived unmet needs of an age-friendly environment: A qualitative exploration of older adults’ perspectives in a Malaysian city

**DOI:** 10.1371/journal.pone.0286638

**Published:** 2023-06-06

**Authors:** Xin-Jie Lim, Chii-Chii Chew, Chee-Tao Chang, Premaa Supramaniam, Lay-Ming Ding, Philip Rajan Devesahayam, Lee-Lan Low

**Affiliations:** 1 Clinical Research Centre, Hospital Raja Permaisuri Bainun, Ministry of Health, Ipoh, Perak, Malaysia; 2 Discipline of Clinical Pharmacy, School of Pharmaceutical Sciences, Universiti Sains Malaysia, Minden, Penang, Malaysia; 3 School of Pharmacy, Monash University Malaysia, Subang Jaya, Malaysia; 4 Perak State Health Department, Ministry of Health, Ipoh, Perak, Malaysia; 5 Institute for Health Systems Research, Ministry of Health, Setia Alam, Shah Alam, Selangor, Malaysia; Al Mansour University College-Baghdad-Iraq, IRAQ

## Abstract

This exploratory qualitative study investigates older adults’ unmet needs in the age-friendly city of Ipoh, Malaysia. Seventeen participants were interviewed, including ten older adults residing in Ipoh City for at least six months, four carers, and three professional key informants. Interviews were conducted using semi-structured questions based on the WHO Age-Friendly Cities Framework. A 5P framework for active ageing based on the ecological ageing model was adapted for data analysis. The 5P framework consists of domains of person (micro), process (meso), place (macro), policymaking (macro), and prime, which allows for the dissection of older adults’ unmet needs in planning for multilevel approaches, which were employed for analysis. **Person**: the personal needs requiring improvement included digital divide disparity, inadequate family support, and restricted sports activities attributed to physical limitations. **Process**: There were fewer social activities and a lack of low-cost and easily accessible venues for seniors. Economic challenges include expensive private healthcare services, variation in the quality of care in older residential care facilities, and limited savings for retirement. **Place** issues include unequal distribution of exercise equipment, public open spaces, the need for more conducive parking for seniors, and a place for social activities. Difficulties assessing public transportation, digitalized services, and unaffordable e-hailing services are common among seniors. Housing issues for seniors include a lack of barrier-free housing design and unaffordable housing. **Policymaking**: Insufficient private sector commitment to improving services to older adults, lack of policy governance on the quality of nursing homes, and insufficient multidisciplinary governance collaboration. **Prime**: Health promotion for preventing age-related illness is required to preserve health in old age, and full-time family caregivers’ psychological well-being is often overlooked.

## Introduction

Globally, the number of people aged 60 and above is set to double from 1 billion to 2.1 billion between 2019 and 2050 [1, 2]. In Malaysia, 11.2% of the 33.6 million population aged 60 and over in 2021 [3] will increase to 14.5% by 2040 [4]. Ipoh, Malaysia’s third-largest city with the highest number of older people [5], recognized the critical need for an age-inclusive city and joined the Global Network for Age-friendly Cities and Communities (GNAFCC) in 2018 to initiate the changes [6].

The World Health Organization (WHO) established the GNAFCC in 2010 to unite cities, communities, and organizations to build conducive places for ageing. Within this network, a framework that determines age-friendly cities is described by eight domains: outdoor spaces and buildings, transportation, housing, social participation, respect and social inclusion, civic participation and employment, communication and information, community, and health care. Having these domains addressed in planning a city’s structures and services would promote the development of age-friendly cities and communities in urban life [7]. However, these domains could be too broad for local policymakers to implement specific interventions. Besides, these domains are not independent and may be interconnected.

Developing age-friendly cities goes beyond striving to attain specific goals or pre-determined outcomes. A more inclusive and intersectional approach with implications for policymakers, urban planners, and health professionals should be considered [8]. It should be able to adapt and alter efficiently to sustain the structure of age-friendly cities and ultimately answer the unmet need of older adults. This fundamental element can be seen in the ecological concept. This concept views ageing as a dynamic interaction between an individual and adaptability to the physical and social environment [9]. The principles of the ecological system manifest systemically at all levels, allowing researchers to dissect older adults’ experiences into microsystems, mesosystem, and macrosystems [10, 11]. In addition, the ecological concept is envisioned as nested structures surrounded by and inside one another that allows us to examine the interaction and interplay structure between the layers adapting to the real situation to promote an age-friendly city [12].

An integrated structure between the WHO GNAFCC framework and ecological model not only addresses the requirement of creating an age-friendly city but can provide a more comprehensive understanding of the unmet need of older adults, and designing measures tackles the issues from the core component basing it on the ecological concept of ageing [10, 13]. This can be achieved by adapting the pillars of the WHO GNAFCC that functions as a main guide in elucidating the experiences and needs of older adults for an age-friendly city while the incorporation of the ecological model of ageing with the multidimensional concepts enables multifaceted intervention to be carried out simultaneously.

This study aims to explore the perceived unmet needs of older people based on the perspectives of the ecological ageing concept and, at the same time, fulfilling the requirement of the WHO GNAFCC in creating an age-friendly city by using Ipoh city as a study model. The opinions of key informants, including caregivers and healthcare professionals, in the context of an age-friendly city are explored.

## Materials and methods

### Design & study population

This was an exploratory qualitative study conducted between August and November 2021. Respondents were 60 and above, Malaysian, residing in Ipoh City for at least six months. The cut-off of 6 months is based on the fact that most older adults experienced psychological changes within the first six months of stay at a new place; the change tended to level off after about six months. However, the changes’ duration varies depending on the person’s medical and psychological health [14, 15]. The potential respondents were purposively selected based on their employment status (employed and retired), ethnicity (Malay, Chinese, and Indian), and income (high, middle, and low) in order to obtain a variety of data. These respondents were identified from a pool of participants who agreed to be contacted for in-depth interview (IDI) when they responded to a large-scale population survey study for aged city preferences [16].

Caregivers of older adults and professional key-informants were invited to give their opinions to triangulate the findings from the older adults. We interviewed family caregivers, volunteer caregivers from non-profit organizations (NGO), and paid caregivers from nursing homes. Paid caregivers from nursing homes were identified via recommendations by healthcare providers, while those from NGOs were found through websites and contacted by telephone calls. Professional key-informants, including clinical specialists, geriatricians, and government officials, were identified upon completion of the analysis of IDI with older adults and caregivers.

### Instrument

The development of an interview guide for older adults was guided by the WHO GNAFCC eight domains (transportation, housing, outdoor spaces and buildings, social participation, respect and inclusion, civic engagement and employment, community support and health services, and communications and information) [13]. The content of the semi-structured questionnaire was then confirmed with literature and expert opinions, including geriatricians and officers of the State Health Department and Ipoh City Council. The interview guide was initially created in English and then translated by native speakers of Malay and Chinese.

In this study, "perceived unmet needs" are defined as the gap between an individual’s self-assessment of long-term care needs and the actual resources available to address those needs. The unmet need assessment is not limited to self-report, but also caregivers’ or providers’, and care-recipients’ perspectives on perceived unmet requirements for services and the environment for ageing [17–19]. The interview guide for caregivers and professional key-informants was developed based on issues identified by older adults. The questions were structured with the aim of triangulating the perspectives of older adults with the opinions of caregivers and key informants, as well as gauging the unmet needs of older adults for healthy ageing.

### Data collection

We approached consented respondents in the previous questionnaire survey to seek their interest and agreement to participate in the IDI. Interviews were conducted virtually via conventional telephone calls, through a cloud-based video communication platform without requesting respondents to turn on their video (Zoom^Ⓒ^) or face-to-face based on the respondents’ preference, in view of the COVID-19 pandemic. Each interview session was conducted by two female interviewers (XJL-medical doctor, MSc. PH, and CCC-pharmacist, MSc. both had at least 3 years of experience in qualitative research and were full-time researchers at the time of the study). The respondents were not known to the interviewers. Prior to the IDI, all respondents were informed regarding the purpose of the study and given verbal consent to participate. With permission from the respondents, audio recordings took place during the interview sessions to facilitate transcription and data analysis. All IDIs were conducted virtually, except for one conducted face-to-face, which took place in a private room of the hospital facility where the interviewers worked. Each IDI session lasted approximately 30 minutes to an hour without any repetition of IDI; any information gaps were probed in a subsequent interview with another respondent. None of the respondents was contacted again for new opinions. The transcripts were not returned to the respondents for comment, and memo were taken during the interview to facilitate debriefings between the interviewers at the end of each IDI, and the information gap requiring further exploration were highlighted for the next IDI. The interview guide for older adults was revised following the preliminary analysis. The data collection among the older adults was stopped once saturation was achieved at the seventh older adult, whereby the coders identified no new information. Three additional older adults were invited for the IDI as a confirmatory measure to ensure no new emerging information was found. The findings were then discussed further though IDI with the key informants.

### Data analysis

In identifying ecological model of ageing, a framework—the 5P framework for active ageing—that takes into consideration the ecological concept by considering the preferences for ageing from a personal, spatial, socioeconomic, governance, and health perspectives was selected to guide the data analysis [10, 20]. The 5P framework adapted the ecological approach of ageing that consists of micro, meso, and macro dimensions and further divided into five layers: "**P**erson" (micro), "**P**rocess" (meso), "**P**lace" and "**P**olicymaking" (macro), and "**P**rime". This framework specifically emphasizes the inter-relationship between the 5P layers [20]. This framework is appropriate to be used in analysing the unmet needs of ageing as it takes into consideration of WHO’s eight domains of ageing [13], as well as incorporates the interconnectedness of individual layers of 5P and demonstrates the relationship between a person and the environment required for ageing. This approach allows various levels of interaction between the environment, human situations, and their relationships.

The data were managed using Microsoft Excel© and Atlas.ti version 9. Each completed IDI was immediately transcribed and analysed by a pair of data coders (XJL, CCC, and CTC-pharmacist, MSc, and PS-researcher, BSc.), and the results were analysed separately. A series of discussions over the findings were discussed among the interviewers, coders, and a senior qualitative researcher (LLL- medical anthropologist, Ph.D). Should any disagreements be raised during the discussion, the opinion of the senior researcher was sought, and a literature search was performed to justify the coding. A consensus was achieved over the finalized results. The interviews that were done in Malay were transcribed without translation. The analysis was carried out in both English and Malay. The transcripts were read in its original language before being coded in English. For the purpose of publishing, the Malay quotes were translated into English.

Triangulating different sources of information, inter-coder agreement, analyzing and resolving disconfirming data, and supervision from a senior qualitative researcher all contributed to the findings’ credibility.

### Ethics approval and consent to participate

Ethical approval for this study was obtained from the Medical Research and Ethics Committee (MREC), Ministry of Health Malaysia [KKM/NIHSEC/P20-217 (7)]. The study was also registered with the National Medical Research Registry under the protocol number: NMRR-19-3191-51748 (IIR). Informed consent from the respondents was obtain prior to data collection.

## Results

### Demography

A total of 17 respondents, including 10 older adults, four caregivers, and three key-informants participated. The median age of older adults was 68.0 (IQR: 6.5), with six (60.0%) females. The majority of them were retired (60.0%) and had a low monthly income (60.0%). One of them was found staying alone. A paid caregiver, a volunteer caregiver, and two family caregivers participated. The key informants were comprised of a geriatrician, a rehabilitation specialist, and a social security officer from an organization that provides protection to employees and their dependents based on joint responsibility through the pooling of resources, sharing of risk, and replacement of income [21] (Table 1).

**Table 1:** Demographic characteristics of respondents.

Respondent	Gender	Age (year)	Employment status	Ethnicity	Monthly income of older adults (Low/Middle/High)	Staying with	Duration living in Ipoh (year)
**P01**	Female	78	Retired	Chinese	Middle	Family	65
**P02**	Female	69	Retired	Chinese	Low	Family	15
**P03**	Male	73	Retired	Chinese	Low	Family	60
**P04**	Female	63	Retired	Chinese	Middle	Family	38
**P05**	Male	69	Self-employed	Indian	Middle	Family	69
**P06**	Male	71	Retired	Indian	High	Family	68
**P07**	Female	67	Self-employed	Malay	Low	Alone	20
**P08**	Male	61	Retired	Malay	Low	Family	39
**P09**	Female	67	Unemployed	Malay	Low	Family	30
**P10**	Female	63	Unemployed	Malay	Low	Family	14
**CGP1**	Female	60	Private employee	Chinese	NA	NA	27
**CGP2**	Male	53	Private employee	Indian	NA	NA	30
**CGF3**	Female	60	Private employee	Chinese	NA	NA	30
**CGF4**	Male	63	Private employee	Indian	NA	NA	38
**K01**	Male	41	Public employee	Chinese	NA	NA	NA
**K02**	Male	47	Public employee	Chinese	NA	NA	NA
**K03**	Male	41	Federal statutory body employee	Malay	NA	NA	NA

P: respondent, CGP1: Paid caregiver, CGP2: volunteer caregivers, CGF: Family caregiver, K: Key-informant, NA: Not applicable

### 5P ecological model of ageing

The respondents’ perspectives of unmet needs for age-friendly environments were grouped into five domains and 13 sub-domains of the 5P Ecological Model of Ageing (5P model). Table 2 summarises the findings of unmet needs and includes selected quotes from respondents.

**Table 2:** The 5P ecological model of ageing summarizes the unmet need for ageing.

Domains	Sub-Domains	Unmet Need	Quotes
**Person**	**Personal characteristic**	**Digital divide** • Ability to learn information technology • Limited internet access • Magnified digital needs during COVID-19	• We don’t know much about IT (information technology). . . Of course, we can (join online classes), but not at such a high cost. I don’t think the older people can afford to learn IT and then attend classes if it comes at a high cost. . . (P06) • If their children refused to pay for an internet subscription and they (the older people) did not have a pension (retirement fund), it would be a huge burden. . . (P01) • During COVID (COVID-19), when I did not have the WIFI, I really appreciated the 1GB given by the Telco last time. (P02)
		**Family support** • Geriatric care knowledge • Negligence • Older adults staying alone and requiring assistance from relatives.	• Some families visit doctors and don’t even know what a commode chair is, so they just know that if (the senior) can’t go to the bathroom, you just (help the older person to) wear pampers. (K01) • Some of them (the older person) are completely ignored by their family members. . . One of our residents was a psychiatric patient. "None of them are capable of caring for her." (Doctor: "You should invite family members to the next appointment. . .") So, I relayed the message to the family members, but no one showed up. (CGP2) • I am staying alone; I will go to my nephew’s house at night while staying alone during the day. Whenever I have knee pain, I ask my nephew, who stays opposite my house, for help. (P07)
	**Behaviour**	**Health behaviour** • Limited choice of sport activities	I used to play badminton, but now I don’t do anything. Basically, I was unable to proceed due to physical limitations. (Interviewer: What types of sports or facilities are required for the older person to improve their mobility?) Perhaps, as you know, the Chinese art of Tai Chi and other (activities) can make them dance and enjoy themselves. (P05)
**Process**	**Social Environment**	**Social interaction** • Fewer opportunities to engage with youngsters	The younger generations are unwilling to participate in society (tasks), we (the elders) are doing it. They don’t have time, and they’re interested with making money. (P06)
		**Affordability** • Affordable location accessible to seniors for social activities	I didn’t get a good location; I had to climb these stairs. Earlier, I had several older individuals with me, but they had left. They (the older person) are unable to climb the stairs. We are now renting a space upstairs that is a little less expensive for our organization because the downstairs location is too expensive to rent. (P06)
		**Social inclusion** • Tailored community programme for seniors with disabilities	I believe that it is sometimes difficult to handle these (bed- or wheelchair-bound) persons (in terms of participation in activities). . .The local community is supposed to have a self-initiated and self-sustaining body. It is a little more challenging in Malaysia since you need someone from the local community to lead and run things for you. We (professionals) can only provide guidance (due to a shortage of staff). (K02)
	**Cultural Environment**	**The sense of place** • Preferred to age at home • A well-kept city	• I preferred to stay at home when growing old. I prefer staying at home than going there (nursing home), and feeling scared and lonely. I’m not going to feel at ease. It’s not my house. That is why I dislike it (P01) • I remember, wishing I could go back to the good old days when everything was so nicely done. I grew up in this town. It’s rather nostalgic. That was back in the day, when everything was perfectly kept. Now it’s the exact opposite. (P03)
	**Economic Environment**	**Perceived costly private healthcare services**	Healthcare is now so expensive if (you) go to private (healthcare services), which I understand is (that is considered) a very high healthcare cost for young (people), not just older adults. (P05)
		**Health insurance** • Unaffordable • Ineligible due to old age	• At this age, we are not entitled to buy insurance. I knew nothing about insurance (for my age). They don’t think you’re credible (are eligible to purchase). . . They will not accept my age. (P03) • "No," they (insurance agent) told me, “You are already above the age of 70. No, you do not qualify. There is a cut-off point.” (P03)
		**Older residential care facility** • Perceived expensive private nursing home • Perceived sub-optimal quality of older residential home • Perceived lack of government funded day-care centre and costly private centre.	• (Nursing Home) has to be within an affordable range. Most are expensive. In this country, private nursing homes are driven by profit. (P06) • Some long-term care facilities are good; others are bad. Some (older persons) were subjected to horrific treatment, particularly when they were unable to communicate themselves or were paralyzed. . . (P05) • There is a private (day-care centre), but the family must pay a few hundred ringgits to go there. However, some families are unable to pay. Actually, it should be free, and the government should provide them with the necessary facilities … there are private ones where you must pay for the service. However, how many individuals can afford private services? (CGP1)
		**Economic security** • Inadequate saving for retirement	I receive a small pension each month, which is clearly insufficient for me. That is one of the main reasons I must continue working on it. (P05)
		**Employment** • Continue as self-employment for income and passion • Continue employment for self-supporting • Physical limitation • Preference of employer	• There is no such thing as time (for retirement) when you are self-employed, and you know that as long as you can perform work, you can earn money, right? It is, in fact, my passion (too). (P05) • I don’t work (physically). I can only go (to the site) and check first, and then I will know what to do. I get the materials, and then I take my assistant along, and they will carry out the work. But I can’t (work) physically. I can’t work already. But I can drive. Of course, I will assist them (the assistant). (P05) • I did not work (all this while); I was at home only, previously taking care of my mother (and now) she passed away; I became a babysitter because I needed income. I am not physically able to walk and sometimes I am tired (or) having bodily pain. In one to two years (I think), I cannot (babysit) already. (P07) • Most employers don’t want workers at this age (older adults). If they were hiring general workers, they wanted young workers. They will not keep workers in old age. (K03)
**Place**	**Land- use**	**Exercise, sports and recreation facilities** • Inadequate exercise equipment • Unequal distribution of recreational facilities	• No, no, there’s nothing to exercise on, it’s just an empty field, whereas I see Singapore, they’ve got bars, string, (and) everything, you know, a lot of things for you to do. When you want to stretch, you wonder what you want to hold on to stretch. (P04) • They installed those iron bars (for exercise). . . (in) a neighbourhood where the wealthier people. . . In my area, we have a lot more of those poorer parts of society. . . I wish I had those assets. The grass is not well attended to. We can’t even use the field. You can’t even play football with your grandchildren. (P03)
		**Public Facilities** • Not conducive parking place	There is not much of a conducive parking area. I don’t think the older adults are all in good health. If the parking areas are reserved for the older adults, we can park nearby and walk there. But there isn’t such a thing. But (for) OKU (disabled people); Yes, one or two are reserved for OKU parking. (P06)
		**Place for social activity** • Social life is limited by socio-environmental constraints	• I think the government should do something to help the older adults. Let’s say we provide a space for them where they may socialize with the rest of them. Because old folks like conversing with one another. . . That’s why I’m hoping the government would take action, whether by establishing a centre or creating a park nearby. . . (CGF3)
	**Access**	**Connectivity** • Non-optimized bus stop location	If they (older adults) are unable to drive and have no one to assist them, a bus is essential. There must be an older adult-friendly station where they may return and then walk a short distance back to their house or take a bus. (P01)
		**Accessibility to public transport** • Lack of barrier free transport system • Fear of public transportation during COVID-19	• Yes, since if you’re taking the bus, you must be wheelchair or disability body friendly. First of all, the route (and) the pavement must be custom-made. (K02) • In (my) life, I ask for help from my friends and nephew because I do not have money to purchase (transport). I asked for help from my brother or nephew to send me (to the primary care clinic); previously, I could still take a bus, but now, I am (physically) unable to take a bus. (P07) • Now with COVID (COVID-19), I am afraid to take the bus; I used to take a bus, but because I am old, I am afraid to take the bus. (P07)
		**Affordability e-hailing service**	They want us to use Grab, but it’s too expensive for us. It’s convenient, but it’s also expensive. They ask (for) a few dollars. Oh, I’ll just spend these few dollars on breakfast, lunch, and dinner. . . (P03)
		**Accessibility to services** • Marginalized due to digitalization of services • A priority lane for seniors in certain services	• They (the government) may want to be innovative and become cashless, so hopefully you will comply, but you must recognize that many people are still older persons. The old generation is still around. (P03) • The older people had to wait in a long queue to see a doctor. . . "These older adults were fasting (for blood taking) and now is 11 a.m., “Can we (caregivers) skip the line?" They (healthcare providers) told us that we couldn’t (skip the queue) … and there was no priority. They will only entertain you for express service if you are in a wheelchair or using a walking stick. (CGP1)
	**Public open spaces**	**Pedestrian safety** • Combination of audio and visual pedestrian crossing aids • Maintenance of pedestrian crossing aids • Crossing time	• There is no sound, only lightning. They just showed the picture. The green people are walking (sign). Sometimes sound and vision together is better than just visually. (P02) • Some have to press the button (for pedestrian crossing aids); but, when you press the button, the button doesn’t work. They never maintain it. (P03) • (It is) a bit fast. It’s better to make it slower for them to take their time walking on the road. Road crossings, especially for older people, are very slow. You want them to run? It would be very dangerous. . . We must set more time for them. (P01)
		**Maintenance of outdoor space** • Maintenance of lawn and facilities	The fields are not well maintained. They’re not being attended to. . . Chair and bricks have been broken. They just cut the grass, and that’s all… Our drains were not swept. I mean, there’s still the sort of accumulation of, you know, what we call clogs. (P03)
	**Housing**	**Universal design** • Barrier-free concept in house designing	I have a 16-inch wheelchair, but my house is so cluttered that there is only a 10-inch doorway that will allow me to enter. There are two toilets in the house. One is seated and one is squatting, so we removed the squatting toilet and replaced it with the sitting toilet. (K01)
		**Affordability of housing** • Unaffordable housing for older adults	• Because in the first place, the older people won’t be able to get the loan. Either they have their own savings or they can’t buy a house. Because no bank will borrow money to the older people if they don’t have some kind of guarantee. The prices of houses are atrocious. . . So, the question is whether the older adults can afford it or not. (P02) • It is very important (to own a house), but I am not affording to buy a house (as) I do not have source of income. This is because; I have to take care of my mother when I was young, and when my mother passed away, I have to stay alone. (P07)
**Policy making**	**Good Governance**	**Governance and implementation** • Commitment from nursing home for capacity building • Regular monitoring the quality of care in nursing home	• Even if you have a training course, such as a dementia training course, not many nursing homes will come in at the end since there is no added bonus to sending the staff to learn this… (K01). • I cannot find one (a nursing home) that is good enough for me to send my loved ones to. So even if I have money, I can’t find that kind of nursing home that is of quality. It all goes down to no governing. I mean, no one is looking at it (quality of nursing home) as a clear border between what is a nursing home and an old folks’ home. It’s mushrooming everywhere. (K01)
		**Multidisciplinary collaboration** • Integrated geriatric care • Administrative support	• So, what we do here is identify the person. A geriatrician, for example, will serve as the coordinator, so we will hold a multi-disciplinary team meeting. . ., and we will then have an agreed-upon strategy for the patient. So, there are three components to treating an older person: individualized, holistic, and multidisciplinary approaches. (K01) • One barrier is that top management’s understanding of it (geriatric care) is still, you know, inadequate, and having top management understand it (geriatric care) and then support it is important. (K01).
		**Performance orientation** • A sustainable home visit service • Awareness of domiciliary care among healthcare providers	• The B clinic (government primary care clinic) used to have a policy that … they used to come to the nursing home and do the dressing. . . You merely need to call, then go to the clinic and give them the (older people) data… However, this policy is no longer in effect because they claim to be understaffed. (CGP2) • The plan is to monitor for three months before discharging the patient from domiciliary care. . . The (healthcare) team comes to the patient’s house within 48 to 72 hours of discharge. However. . . . Nobody knows what the system is. . . It receives very few referrals. (K01)
		**Inclusiveness** • A more thoughtful design of age-related policies	I still ride a motorcycle and drive a car. There is mobility. I’m not OKU. (a disabled person). It’s ridiculous that senior citizens of a particular age are not allowed to drive, as recently reported in the media (at the time this study took place). So, you instil fear in people like us. There are a lot of people who are against it. Is it we (the older citizens) who are driving against the pedestrian lane and hitting someone? It does not apply to everyone. (P03)
**Prime**	**Physical Health**	**Health promotion** • Early education to prevent age-related disease	Even starting in schools, taking enough calcium and vitamin D would have prevented the 20- to 30-year-old ladies from osteoporosis. Again, nothing on, until 20 years (later) if they get a fracture. How often hearing about aerobic exercises?. . . . They are important for balancing, strengthening, and flexibility. (K01)
	**Mental Health**	**Cognitive functioning & psychological wellbeing** • Knowledge in handling the older adults with cognitive dysfunction • Respite care to accommodate the psychological wellbeing of family caregivers	• One example is a 70-plus (older adult) resident’s stay in a nursing home. Everything started with the shingles, and that triggered the delirium. Nobody is aware that it is (a) possible (cause of delirium). The older people … was diagnosed with dementia. The family panicked …, so they sent the older adult to a nursing home. . . (They) administered antipsychotics to patients. . . I confirmed the delirium through a comprehensive assessment and diagnosis. I provided them (the family) with a discharge plan: "Why don’t you try bringing him (the older adult) back home for a one-month trial period?" “Give him your support." … Within two months, the patient (the older adult) had recovered completely. . . and was driving back to the community. (K01) • It is not the popular respite care, like giving the caregivers a break, you know? They (family caregivers) look after their parents and when they want to go for holidays, they need somebody to really keep an eye on their parents. They (family caregivers) said they can’t afford (RM) 500 a day for home private nursing. (CGP1)
	**Social Health**	**Lack of affordable social activities for senior citizen**	Actually, once in a while, I think yes (to have social activities). They should go out with the family members or do things like go to the theatre and so on. . . I probably do it once a month. That is not an easy task. And it’s sometimes nice to have some fun (entertainment activities) while also getting a discount. (P06)

### Person

The theme "person" refers to the impacts of personal characteristics and behaviours on ageing [20].

#### 1.1 Personal characteristics

Personal characteristics in the ageing process encompasses the dimensions of health, age, genes, educational level and socio-economic status, family background and self-efficacy [20]. Lower socio-economic status and inadequacy of family support were identified in the prospect of personal needs in this study.

"Digital divide," defined as unequal access to the internet for activities, was highlighted for the older adult in the lower socio-economic status [22]. Digital illiteracy and the high cost of internet usage limit their opportunity to internet access. The digital need among older adults was magnified during COVID-19 among those without internet access before the pandemic. The respondents appreciated the telco company’s free internet access of up to 1GB.

Family support is essential for older adults, ranging from direct care to complex health care and social service [23]. This support is crucial for those undergoing rehabilitation or residing in nursing homes. However, the current healthcare system does not provide adequate training for family members to continue geriatric care at home. Those who reside in a nursing home are often neglected by their family. Older adults who live alone face physical deterioration, lack skills, and live with limited incomes, requiring assistance accessing food, health, and essential daily services. This study found that family support is not limited to direct family members; extended family members such as relatives were important in assisting with the basic needs of older adults living alone.

#### 1.2 Behaviour

A healthy lifestyle with adequate physical activity is vital for older people’s physical well-being to sustain healthy ageing [20]. Older adults and caregivers recognize the importance of physical activity, but their activities, especially in sports, are constrained by age. However, older adults can identify activities, such as tai-chi or dancing, that are age appropriate and can get pleasure at the same time.

### Process

Social, economic, and cultural environments are essential elements in creating barrier-free daily activities for the ageing process [20].

#### 2.1 Social environment

Independent, activity, coherence, peer support, and social networking are the fundamentals of a healthy social environment [24]. A lack of engagement by youngsters, unaffordable venues, and a lack of inclusion for wheelchair-bound older adults can reduce the interest of older adults in social activities. Their physical, financial, digital, and environmental requirements are factors to be considered to facilitate their participation in social programmes.

#### 2.2 Cultural environment

The cultural environment is an important factor in how people make sense of a place by integrating physical forms [25]. Older adults are more likely to identify a place within their communities, enabling them to continue living in the environment they are familiar with [26]. In general, our respondents preferred to age in their own house due to a secure feeling.

The quality of the exterior environment, service quality, and place dependency are all important factors in a place’s uniqueness [26]. In the neighbourhood, the respondents claimed that the quality of services and infrastructure has been decreasing over time with poor maintenance, leading to inadequacy of leisure spots.

#### 2.3 Economic environment

Health care services, insurance coverage, pensions, socio-economic status, and employment were the context of the economic environment.

Malaysian citizens can choose to obtain their health services from a government-funded public arm or a pay-per-use private arm, and the population aged ≥ 60 is free of charge for public healthcare services [27]. On the other hand, the older adult perceived that the healthcare cost of the private sector was high and unaffordable without regard to the age group.

Research shows that insurance coverage that offers more comprehensive, accessible, and affordable coverage for healthcare services would increase the utilization of healthcare services [27]. Even though older adults in this country are offered free access to public healthcare, a study found that people are more willing to utilize public healthcare services despite holding private health insurance coverage [28]. People with health insurance can use healthcare services more appropriately and associated with better health outcomes [29]. However, the respondents in this study perceived that only a small proportion of Malaysians purchased health insurance and the current insurance policy limited eligibility due to a predetermined age limit or financial incapability.

Older adults demonstrated different preferences in their long-term care centres, which may have influenced their long-term health outcomes [30]. The respondents identified several care facilities for older adults, such as nursing homes, old folks, and day-care centres. Nursing homes were perceived to be costly and unfavourable for healthy ageing, while older adults may be put at risk due to the quality of care. Day-care centres were perceived to be expensive and out of reach for low-income families. Some of the caregivers in this study deemed that the government should subsidise day-care centres services among financially incapable families.

Older adults are motivated to continue working due to limited pensions and retirement savings, allowing them to self-sustain without relying on others financially. Physical limitations associated with ageing and employment policies, such as the retirement age, affect employment opportunities. Besides, a key informant pointed out that employers prefer young workers and are unlikely to continue to employ older adults even though they are suitable for that position. Continuing employment was vital for older adults who were alone without a direct family member to support them in sustaining their needs. Financial assistance from the government was a medium to reduce their financial burden.

### Place

The theme of "place" refers to environmental features that encourage active ageing and improve senior well-being, which are comprised of optimal land use, good accessibility, safe public open spaces, and age-friendly housing modifications.

#### 3.1 Land use

Land use is defined as the diversity, quality, composition, and arrangement of amenities and facilities such as recreational parks, parking spaces, markets, aged-care facilities, banks, post offices, health clinics in a neighbourhood [20].

Older adults need recreational parks to exercise and socialize, and outdoor fitness equipment is becoming increasingly popular in Asian countries. However, some respondents raised that there was an unequal distribution of recreational facilities, especially in neighbourhoods with lower socio-economic residents. Family caregivers highlighted the lack of outdoor places for older adults with disabilities and suggested a centre or space allow social interaction.

Older adults who have experienced physical function changes may face difficulties encountering narrow parking spaces. They proposed that parking spaces be made broad enough to reduce the risk of collision and provide parking spaces in the vicinity of the desired destination for older people.

#### 3.2 Public open spaces

Public open spaces encompass safe pedestrian crossings and adequate maintenance of the landscape [20].

The respondents pointed out that, older adults require longer crossing times at intersections. Providing a shorter crossing distance or a longer green interval is crucial for safe crossing among the older population. Regularly maintaining the crossing aids was instrumental in ensuring a safe road crossing.

The respondents reiterated regular and properly maintained lawn and outdoor facilities. They emphasized that administrators should ensure regular mowing and scheduled repair of broken park equipment when the current amenities of the parks are currently not well maintained.

#### 3.3 Access

Access is defined as the connectivity to different areas in a community, the availability of different routes, street infrastructure, and public transportation options [20].

The respondents deemed that public transportation is essential for older adults, especially when they have lost the ability to drive and those staying alone without the financial ability to purchase a vehicle. They suggested that the bus stops should be located close to the housing areas, with safe pavement conditions to improve walkability. Bus design should meet the needs of older people, with low-floor entrances, handrails, priority seats, and wheelchair space. The issue of public transport was exacerbated during COVID-19, with one of the main reasons being fear, besides the age factor. Some respondents felt that e-hailing is more convenient than other modes of public transport, but the cost outweighs the convenience.

Access should extend beyond physical connections and include a range of digital services. The digital services should be tailored towards accessibility for older adults who may not be as technologically savvy. Besides, some respondents noticed that priority lanes for seniors in obtaining services are not provided in all the service areas, and priority is only given to consumers who are physically disabled, even though the older adults have the needs.

#### 3.4 Housing

Neighbourhood safety, home support interventions, quality residential care facilities, universal design of housing, and affordable housing are all important aspects of age-friendly housing [20].

In key informants’ opinions, home modifications such as residential grab bar installations, conversion of squat-to-sit toilets, construction of an attached bathroom, and wide door steps for wheelchair entry were some safety features proposed to accommodate older adults’ needs. However, home safety measures to facilitate ageing is lacking, with many older adults unable to afford a house due to financial, psychological, or technical barriers. Owning a home was perceived as necessary but limited by the realistic conditions in which it was unaffordable for the group of older adults without technical skills, living alone, and not earning sufficient incomes to secure a bank loan in house purchasing.

### Policymaking

Tolerance, fairness, social justice, and good governance are key concepts for sustaining urban planning development for an age-friendly city [20]. Five subthemes are governance and implementation, multidisciplinary collaboration, performance orientation, openness, transparency, integrity, and inclusiveness.

#### 4.1 Governance and implementation

A well-planned policy design requires network governance, in which government activity is linked to the private sector and civil society players in order to aggregate recipients’ actual needs [31].

Policies promoting an age-friendly city were perceived as lacking and received inadequate attention from stakeholders, such as caregivers of nursing homes. Some of the current policies allow the private sector to operate older adult care services, but their implementation may not be adequately monitored by governing bodies and may result in inconsistent service quality for older people

#### 4.2 Multidisciplinary collaboration

Network governance emphasizes the importance of organic, collaborative structures for active ageing [31, 32]. Inadequate collaboration among multi-disciplinary members is a major barrier to successfully implementing integrated geriatric care. Collaboration can be achieved through a coordinator and administrative support from the top management to eliminate barriers to multi-disciplinary collaboration.

#### 4.3 Performance orientation

The governance paradigm must consider the expert opinion, people’s perceptions, and experience. The governance model must be public policy to engage multi-stakeholder partnerships to ensure successful ageing city development [31, 33].

Inadequate governance commitment in strategies and operation of policies can lead to the termination of age-friendly policies. Meanwhile, the non-performance of policies is also evident in the domiciliary care service—a home-based rehabilitation program to ensure continuity of care at home—lacked coordinated referral mechanisms.

#### 4.3 Inclusiveness

Inclusiveness is a key principle of good governance, promoting fairness and a fair distribution of decision-making authority [34]. Policies should consider older adults’ basic needs and be more comprehensive before implementation. Policies should consider older adults’ basic needs and be more comprehensive before implementation. For instance, the issue involving motor vehicle license issuance to older adults is complex. Age is not the sole factor but the older adult’s physical, cognitive, and functional abilities. Furthermore, that requires a comprehensive discussion of all stakeholders to exert fair distribution for an age-related policy.

### Prime

Prime represents any factors and components that influence the health of older adults. Within this context, the “prime” theme consists of physical health, mental health and social health.

#### 5.1 Physical health

One of the major determinants of physical health includes health promotion as a preventive measure for age-related disease [35]. Additionally, healthcare professionals play an important role in promoting lifestyle factors, such as routine exercise and dietary supplements, to improve physical health and prevent age-related bone loss and osteoporosis.

#### 5.2 Mental health

Older adults’ cognitive functioning and psychological well-being are essential components of mental health [20]. Mental illness is prevalent among nursing home residents. Older adults with dementia, major depressive disorder, delirium, and other psychotic disorders may face challenges due to inadequate knowledge of geriatric care among healthcare providers, nursing home caregivers, and family members. A better system is needed, including specialized nurse training, appropriate use of psychotropic medications, and geriatric care in handling the unspoken needs of older residents to ensure timely actions and referrals. The psychological wellbeing of family caregivers who provide long-term care for older adults at home has not been adequately addressed by the health policymakers. This was due to the lack of the respite care concept being introduced to the local population.

#### 5.3 Social health

Social engagement is a major determinant of social health as it offers older adults a sense of identity and fulfilment [20]. Engagement in activities, such as family activities and leisure activities, can foster interpersonal interactions. However, the activities may not be affordable for older adults, especially when there is no privilege or discount for them in obtaining the entertainment.

## Discussion

This qualitative study explored the perceived unmet needs of older adults of an aged-friendly city based on the 5P ecological model of ageing (5P model). The model depicts micro, meso, and macro dimensions based on health environment and explains the dynamic interaction of these dimensions through the lens of ecological concept [20]. The ageing population should be given the resources to maintain a balance of physical and mental health at the micro level including personal characteristic, social well-being, and spirituality, transcendence at the meso level, while living in a favourable environment at the macro level which involving policy making. The findings of this study helped to define and guide town-planning policies and implementations, and the interconnections between the five dimensions of ageing would allow for repercussions in almost all other dimensions if any of the dimensions were to be intervened in.

### Microsystem

The micro aspect of the 5P model focuses on a person’s physical characteristics, resources, patterns of activity, roles, and interpersonal relationships to improve their digital literacy and address personal needs [22, 36]. Digital access was a mounting concern by the majority of respondents in terms of unmet personal requirements. Prioritising digital skills training for older adults with decentralisation and targeted training at different levels. Additionally, an integrated system with collaboration among government and non-government organizations with internet providers should be sought to eliminate gaps in internet affordability and accessibility for seniors. Low-cost internet access for seniors, even if they are on a fixed income, is advocated for in the U.S.A, as it can help them develop digital citizenship and eliminate social exclusion [37, 38].

Family support and caregiving are vital, it has a direct and ongoing impact on an older adult’s everyday life. This study showed that family caregivers are frequently underequipped and lack the specialised knowledge and skills in caregiving. A protected transition period prior to discharge should be reserved for the caregiver to allow for a structured training session. Necessary aids for family caregivers, including respite care services, should be provided to allow short-term relief from stress [39]. Community health care experts can assess needs, provide information, generate referrals, and integrate care into a larger support plan [40].

Physical activities have a direct effect on the likelihood of healthy ageing, but there is a negative relationship between age and sports engagement [41, 42]. Poor outdoor exercise equipment is associated with sports needs for ageing; therefore, tailored exercise prescriptions should be tailored to age-specific needs. Preserving seniors’ range of movement and muscle strength is key to improving their locomotion ability and functional independence [43], upper body muscular strength and physical functions [44]. It is important to allocating resources with a variety of stakeholders, including healthcare providers, community groups, and commercial enterprises, as well as customising activities tailored to the functional needs and physical abilities of older adults.

### Mesosystem

The mesosystem in the ecology of ageing describes the interrelationships between two or more major settings, such as health care and retirement [45]. Insufficient savings can lead to catastrophic health expenditures due to insufficient income and lack of medical insurance. Social welfare services are often the safety net, but funding is minimal and fragmented [46]. Moreover, the existing insurance system does not cover outpatient treatment or pharmaceutical costs, leaving older adults or their families to shoulder the out-of-pocket healthcare costs [47]. The pre-determined age limit of insurance purchase and coverage was a concern the respondents raised when the interviews took place in 2021. In 2023, a private insurance company raised the coverage period to 100 years old, with the maximum entry age set at 70 years old [48].

The Minimum Retirement Age Act 2012 sets the retirement age at 60 [45]; it is suggested to increase the retirement and re-employment age to 65 and 70 to support older citizens who wish to continue working [49]. If a higher mandatory retirement age was implemented, there is a concern that the job opportunities for the younger generation may be lesser. Nevertheless, study shows that different generations have different expectations when selecting careers [50]. Recruiting seniors as mentors to coach youth in employment could benefit both generations [51].

### Macro system

The macrosystem comprises laws, law enforcement practises, government agencies, political parties, social policies, healthcare resources, and various forms of influence that create the social, political, and financial contexts for development [52]. Although not directly experienced by the older adults, the outer layer of 5P model, or macro system, can have a substantial impact on the microsystem that affects the aged. This study found that well-structured transportation system, housing design policies, home visit services, older adults’ residential homes, domiciliary care, and geriatric care training concerning the macro level are directly affecting the micro needs of the older adults in their daily routine.

Transportation is essential for older people’s independent lifestyle, but age-related constraints can make driving difficult [53, 54]. Older adults were disadvantaged by the long-distance walking to the bus stop and the high bus-boarding step. Malaysia’s rollout of low-floor, non-step buses with ramps accessible by wheel-chair has been slow, and macro-level planning should include efforts to renovate all bus stops with kerbside access, wheelchair-friendly and able to deploy the ramps [55].

Ageing may impair physical and cognitive functions, leading to the need for broader parking spaces and signals for street crossing. Audio-visual signals provide meaningful stimulation, which could be related to walking faster across the street [56, 57]. It is suggested that auditory and visual signals be installed at signal-controlled junctions. This safety feature should be mandated in all town planning. Safe road crossing facilities protect older pedestrians and users from all age groups, including people with disabilities.

Although aged-friendly housing development is paving its way to meet the need of the older community in certain cities, including Ipoh (the study location), the concept of an aged-friendly city should be mandated in all housing development as a measure to consider the future demands of tenants [58]. The home safety features for an older adult should include wider doorways, grab bars, and non-slip flooring. Additionally, aged-friendly neighbourhoods should consider the amenities such as healthcare facilities, fitness centres, and social spaces.

When implementing policies, age-friendly city initiatives, such as the home visit programme, should consider sustainability, promotions, and public competency. Financial resources and human capital are needed to implement these initiatives, particularly for long-term, large-scale endeavours [59]. Domiciliary care service, a home-based rehabilitation, is an effective intervention to reduce mortality [60]. The Malaysian Ministry of Health (MOH) has offered this service since 2016, the implementation has been limited by human resource dependency and a lack of promotion among healthcare personnel [61]. Organisations and coordination from multiple bodies are essential for the sustainable implementation of a service.

### Impact of COVID-19

The COVID-19 pandemic has had a significant impact on the lives of older adults, from personal, environmental and habitual factors, due to the pandemic. These impacts included worsening psychological symptoms, increased loneliness, reduced quality of life, increased depression, service access issues, sleep disturbances, and reduced physical activities [62]. It is understood that older adults’ unmet needs, particularly in terms of personal, social, and domestic prospects, may be more significant than those of pre-pandemic, as experienced by some of our respondents.

### Limitations

Recruitment problems were seen in the pandemic, where research team did not reach out to people who were wheelchair- or bed-bound and lived in nursing homes. Nevertheless, caregivers were invited to the study to gain insight into the needs of these populations. The IDI conducted via telephone limits the opportunity to build good rapport between the interviewer and the respondents. However, the interviewers reassure that the conversations were kept confidential, that it was informal chatting, and that the voice record will be deleted after transcribing the words. This study managed to recruit only one older adult who is staying alone; future studies could explore further aspects this population. The perspective of an older population living in a city for less than ten years needed further research, as all our respondents have lived in this city for more than 10 years.

### Conclusion

Our findings underscore the perceived unmet needs of older people across several dimensions, including micro (person), meso (process), macro (place and policymaking), and health (prime). The broader implications of this study enable each level to be contextualised and explored in its own right. Multilevel planning approaches to address gaps from different levels concurrently or in a novel form of synthesis are likely to be practical and beneficial.
